# *N*-(2-Methyl-3-oxo-1,3-diphenyl­prop­yl)acetamide

**DOI:** 10.1107/S1600536813007320

**Published:** 2013-04-05

**Authors:** Deli Yang, Daxin Shi, Qi Zhang, Hongxin Chai, Jiarong Li

**Affiliations:** a School of Chemical Engineering and Environment, Beijing Institute of Technology, Beijing 100081, People’s Republic of China

## Abstract

In the title compound, C_18_H_19_NO_2_, the dihedral angle between the benzene rings is 42.0 (1)°. In the crystal, mol­ecules are linked by N—H⋯O and C—H⋯π inter­actions.

## Related literature

For the biological properties of *N*-(2-methyl-3-oxo-1,3-diphenyl­prop­yl)acetamide derivatives, see: Barluenga *et al.* (1993 ▶); Casimir *et al.* (1995 ▶) and for their synthesis, see: Dakin & West (1928 ▶); Selvam & Perumal (2009 ▶); Heravi *et al.* (2009 ▶).
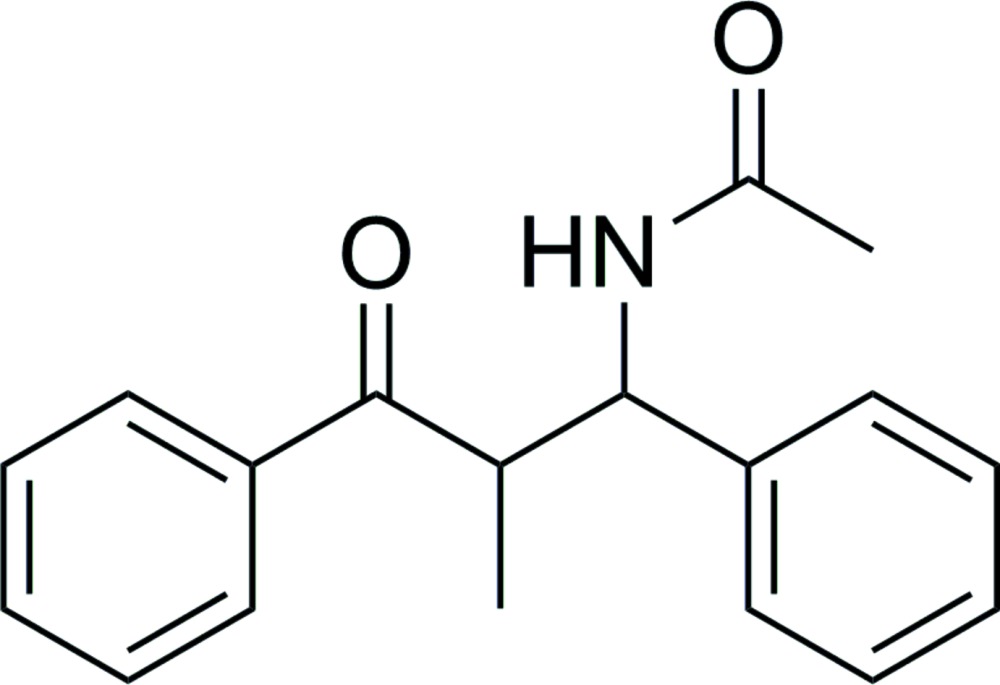


## Experimental

### 

#### Crystal data

C_18_H_19_NO_2_
*M* *_r_* = 281.34
Monoclinic, 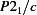

*a* = 9.156 (5) Å
*b* = 17.668 (8) Å
*c* = 10.103 (5) Å
β = 107.914 (7)°
*V* = 1555.0 (13) Å^3^
*Z* = 4
Mo *K*α radiation
μ = 0.08 mm^−1^
*T* = 153 K
0.61 × 0.07 × 0.02 mm

#### Data collection

Rigaku AFC10/Saturn724+ diffractometer
Absorption correction: multi-scan (*CrystalClear*; Rigaku, 2008 ▶) *T*_min_ = 0.954, *T*_max_ = 0.998
12600 measured reflections
3028 independent reflections
2386 reflections with *I* > 2σ(*I*)
*R*_int_ = 0.050

#### Refinement

*R*[*F* ^2^ > 2σ(*F* ^2^)] = 0.070
*wR*(*F* ^2^) = 0.156
*S* = 1.00
3028 reflections
197 parameters
H atoms treated by a mixture of independent and constrained refinement
Δρ_max_ = 0.22 e Å^−3^
Δρ_min_ = −0.24 e Å^−3^

### 

Data collection: *CrystalClear* (Rigaku, 2008 ▶); cell refinement: *CrystalClear*; data reduction: *CrystalClear*; program(s) used to solve structure: *SHELXS97* (Sheldrick, 2008 ▶); program(s) used to refine structure: *SHELXL97* (Sheldrick, 2008 ▶); molecular graphics: *CrystalStructure* (Rigaku/MSC, 2009 ▶); software used to prepare material for publication: *CrystalStructure*.

## Supplementary Material

Crystal structure: contains datablock(s) I, global. DOI: 10.1107/S1600536813007320/lx2279sup1.cif

Structure factors: contains datablock(s) I. DOI: 10.1107/S1600536813007320/lx2279Isup2.hkl

Supplementary material file. DOI: 10.1107/S1600536813007320/lx2279Isup3.cml

Additional supplementary materials:  crystallographic information; 3D view; checkCIF report

## Figures and Tables

**Table 1:** Hydrogen-bond geometry (Å, °) *Cg*1 and *Cg*2 are the centroids of the C10–C15 and C1–C6 benzene rings, respectively.

*D*—H⋯*A*	*D*—H	H⋯*A*	*D*⋯*A*	*D*—H⋯*A*
N1—H1*N*⋯O2^i^	0.94 (3)	1.98 (3)	2.874 (3)	158 (2)
C1—H1⋯*Cg*1^i^	0.95	2.85 (1)	3.649 (3)	142 (1)
C16—H16*A*⋯*Cg*2^ii^	0.98	2.98 (1)	3.472 (3)	112 (1)

Symmetry codes: (i) 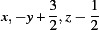; (ii) 
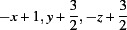
.

**Fig. 1.**
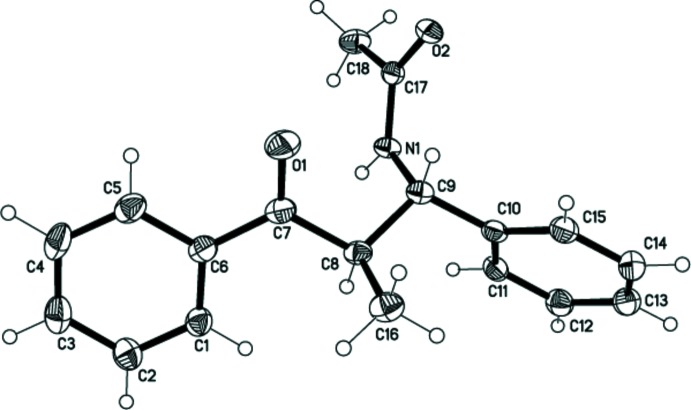
The molecular structure of the title compound with the atom numbering scheme. Displacement ellipsoids are drawn at the 30% probability level.

**Fig. 2.**
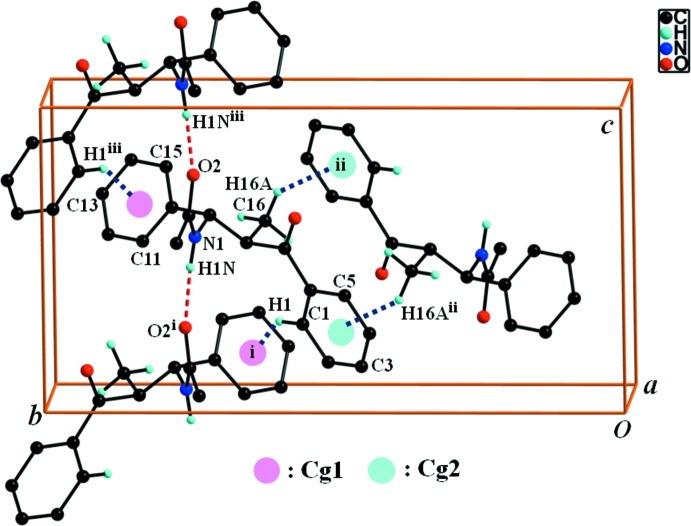
A view of N—H···O and C—H···π interactions. (dotted lines) in the crystal structure of the title compound. H atoms non–participating in hydrogen–bonding were omitted for clarity. [Symmetry codes: (i) x, - y + 3/2, z - 1/2; (ii) - x + 1, - y + 1, - z + 1; (iii) x, - y + 3/2, z + 1/2.]

## supplementary crystallographic information

### Comment

N–(2–methyl–3–oxo–1,3–diphenylpropyl)acetamide is a class of 2–acetamino carbonyl compounds which exhibit great importance of biological (Casimir *et al.*, 1995) and pharmacological (Barluenga *et al.*, 1993) properties. Here, we report the crystal structure of the title compound. In the title molecule (Fig. 1), the dihedral angle formed by the benzene rings is 42.0°, and the methyl and the acetamide groups have an anti–conformation. In the crystal structure (Fig. 2), molecules are connected by N—H···O and C—H···π interactions (Table 1, Cg1 and Cg2 are the centroids of the C10–C15 benzene ring and the C1–C6 benzene ring, respectively).

### Experimental

A solution of benzaldehyde (2 mmol) and propiophenone (2 mmol) in the presence of acetyl chloride and TiCl_4_ was stirred in acetonitrile (5 ml) at room teperature for 3 h. The reaction mixture was poured to room temperature and then filtered to give the title compound. The product was recrystallizated from petrolum ether and ethyl acetate to give white crystalline powder. m.p. 439–441 K.

### Refinement

C—H were included in the riding model approximation with C—H distances 0.95–1.00 Å, and with U_iso_(H)=1.2U_eq_(C) or 1.5U_eq_(C)(methyl). Freely refined H atoms of NH group were located in difference Fourrier maps with N—H distances 0.94 Å with U_iso_(H)=1.5U_eq_(N).

### Crystal data

**Table:**

C_18_H_19_NO_2_	*F*(000) = 600
*M**_r_* = 281.34	*D*_x_ = 1.202 Mg m^−^^3^
Monoclinic, *P*2_1_/*c*	Melting point = 439–441 K
Hall symbol: -P 2ybc	Mo *K*α radiation, λ = 0.71073 Å
*a* = 9.156 (5) Å	Cell parameters from 3372 reflections
*b* = 17.668 (8) Å	θ = 2.3–29.0°
*c* = 10.103 (5) Å	µ = 0.08 mm^−^^1^
β = 107.914 (7)°	*T* = 153 K
*V* = 1555.0 (13) Å^3^	Prism, colorless
*Z* = 4	0.61 × 0.07 × 0.02 mm

### Data collection

**Table:**

Rigaku AFC10/Saturn724+ diffractometer	3028 independent reflections
Radiation source: Rotating Anode	2386 reflections with *I* > 2σ(*I*)
Graphite monochromator	*R*_int_ = 0.050
Detector resolution: 28.5714 pixels mm^-1^	θ_max_ = 26.0°, θ_min_ = 2.3°
phi and ω scans	*h* = −11→11
Absorption correction: multi-scan (*CrystalClear*; Rigaku, 2008)	*k* = −20→21
*T*_min_ = 0.954, *T*_max_ = 0.998	*l* = −12→12
12600 measured reflections	

### Refinement

**Table:**

Refinement on *F*^2^	Secondary atom site location: difference Fourier map
Least-squares matrix: full	Hydrogen site location: difference Fourier map
*R*[*F*^2^ > 2σ(*F*^2^)] = 0.070	H atoms treated by a mixture of independent and constrained refinement
*wR*(*F*^2^) = 0.156	*w* = 1/[σ^2^(*F*_o_^2^) + (0.0511*P*)^2^ + 1.630*P*] where *P* = (*F*_o_^2^ + 2*F*_c_^2^)/3
*S* = 1.00	(Δ/σ)_max_ < 0.001
3028 reflections	Δρ_max_ = 0.22 e Å^−^^3^
197 parameters	Δρ_min_ = −0.24 e Å^−^^3^
0 restraints	Extinction correction: *SHELXL97* (Sheldrick, 2008), Fc^*^=kFc[1+0.001xFc^2^λ^3^/sin(2θ)]^-1/4^
Primary atom site location: structure-invariant direct methods	Extinction coefficient: 0.014 (2)

### Special details

**Table:**

Experimental. Spectral data: IR (KBr): 3297, 3061, 2980, 1683, 1651, 1544, 1448, 1370, 1208, 1140, 970, 707, 615 cm^-1^; ^1^H–NMR(DMSO,p.p.m.):1.13 (3*H*, d, J = 6.8 Hz C_1_H_3_), 1.85 (3*H*, s, C_1_O_1_C_1_H_3_), 4.00–4.14 (1*H*, m, C_1_H_1_), 5.26 (1*H*, t, J = 11.6 Hz, C_1_H_1_), 7.12 (1*H*, t, J = 6.8 Hz, Benzene-H), 7.22 (2*H*, t, J = 8.0 Hz, Benzene-H), 7.29 (2*H*, d, J = 7.6 Hz, Benzene-H), 7.47 (2*H*, t, J = 8.0 Hz, Benzene-H), 7.58 (1*H*, t, J = 6.8 Hz, Benzene-H), 7.80 (2*H*, t, J = 7.6 Hz, Benzene-H), 8.30 (1*H*, d, J = 9.2 Hz, NH); ESI-MS m/*z*: [*M*+Na]^+^ 304.2.
Geometry. All e.s.d.'s (except the e.s.d. in the dihedral angle between two l.s. planes) are estimated using the full covariance matrix. The cell e.s.d.'s are taken into account individually in the estimation of e.s.d.'s in distances, angles and torsion angles; correlations between e.s.d.'s in cell parameters are only used when they are defined by crystal symmetry. An approximate (isotropic) treatment of cell e.s.d.'s is used for estimating e.s.d.'s involving l.s. planes.
Refinement. Refinement of *F*^2^ against ALL reflections. The weighted *R*-factor *wR* and goodness of fit *S* are based on *F*^2^, conventional *R*-factors *R* are based on *F*, with *F* set to zero for negative *F*^2^. The threshold expression of *F*^2^ > σ(*F*^2^) is used only for calculating *R*-factors(gt) *etc*. and is not relevant to the choice of reflections for refinement. *R*-factors based on *F*^2^ are statistically about twice as large as those based on *F*, and *R*- factors based on ALL data will be even larger.

### Fractional atomic coordinates and isotropic or equivalent isotropic displacement parameters (Å^2^)

**Table:**

	*x*	*y*	*z*	*U*_iso_*/*U*_eq_	
O1	0.2368 (2)	0.56897 (11)	0.6152 (2)	0.0553 (6)	
O2	0.13769 (19)	0.74116 (10)	0.76594 (17)	0.0390 (4)	
N1	0.1849 (2)	0.74059 (11)	0.5589 (2)	0.0305 (5)	
C1	0.2662 (3)	0.55877 (14)	0.2653 (3)	0.0401 (6)	
H1	0.3342	0.5998	0.2680	0.048*	
C2	0.2150 (3)	0.51473 (15)	0.1457 (3)	0.0483 (7)	
H2	0.2490	0.5252	0.0677	0.058*	
C3	0.1143 (3)	0.45551 (16)	0.1410 (3)	0.0535 (8)	
H3	0.0791	0.4255	0.0593	0.064*	
C4	0.0646 (3)	0.43978 (15)	0.2540 (3)	0.0520 (8)	
H4	−0.0055	0.3995	0.2497	0.062*	
C5	0.1172 (3)	0.48288 (15)	0.3731 (3)	0.0456 (7)	
H5	0.0841	0.4714	0.4512	0.055*	
C6	0.2190 (3)	0.54355 (13)	0.3808 (3)	0.0373 (6)	
C7	0.2732 (3)	0.58798 (14)	0.5128 (3)	0.0384 (6)	
C8	0.3799 (3)	0.65579 (13)	0.5217 (2)	0.0324 (5)	
H8	0.3626	0.6769	0.4263	0.039*	
C9	0.3469 (3)	0.71774 (13)	0.6157 (2)	0.0317 (5)	
H9	0.3627	0.6956	0.7101	0.038*	
C10	0.4548 (3)	0.78490 (13)	0.6304 (2)	0.0327 (5)	
C11	0.4362 (3)	0.83580 (13)	0.5211 (3)	0.0369 (6)	
H11	0.3546	0.8289	0.4372	0.044*	
C12	0.5363 (3)	0.89691 (15)	0.5338 (3)	0.0444 (7)	
H12	0.5236	0.9308	0.4580	0.053*	
C13	0.6541 (3)	0.90834 (16)	0.6565 (3)	0.0472 (7)	
H13	0.7209	0.9505	0.6656	0.057*	
C14	0.6741 (3)	0.85819 (16)	0.7654 (3)	0.0470 (7)	
H14	0.7551	0.8657	0.8495	0.056*	
C15	0.5756 (3)	0.79640 (15)	0.7523 (3)	0.0400 (6)	
H15	0.5910	0.7618	0.8273	0.048*	
C16	0.5456 (3)	0.62634 (15)	0.5778 (3)	0.0439 (6)	
H16A	0.5626	0.6044	0.6704	0.053*	
H16B	0.6174	0.6683	0.5838	0.053*	
H16C	0.5626	0.5875	0.5148	0.053*	
C17	0.0921 (3)	0.74884 (12)	0.6372 (2)	0.0304 (5)	
C18	−0.0729 (3)	0.76761 (16)	0.5617 (3)	0.0443 (7)	
H18A	−0.1391	0.7270	0.5764	0.053*	
H18B	−0.0867	0.7728	0.4621	0.053*	
H18C	−0.1003	0.8153	0.5976	0.053*	
H1N	0.143 (3)	0.7487 (15)	0.463 (3)	0.045 (8)*	

### Atomic displacement parameters (Å^2^)

**Table:**

	*U*^11^	*U*^22^	*U*^33^	*U*^12^	*U*^13^	*U*^23^
O1	0.0705 (14)	0.0501 (12)	0.0574 (12)	−0.0110 (10)	0.0374 (11)	0.0049 (9)
O2	0.0429 (10)	0.0491 (11)	0.0273 (9)	−0.0032 (8)	0.0144 (7)	−0.0034 (7)
N1	0.0310 (10)	0.0378 (11)	0.0252 (10)	0.0059 (8)	0.0122 (8)	0.0044 (8)
C1	0.0411 (14)	0.0314 (13)	0.0484 (15)	−0.0016 (11)	0.0149 (12)	−0.0001 (11)
C2	0.0540 (16)	0.0398 (15)	0.0507 (17)	0.0019 (13)	0.0157 (14)	−0.0025 (12)
C3	0.0512 (16)	0.0361 (15)	0.065 (2)	−0.0022 (13)	0.0063 (15)	−0.0054 (13)
C4	0.0422 (15)	0.0315 (14)	0.078 (2)	−0.0072 (12)	0.0126 (15)	0.0000 (13)
C5	0.0415 (15)	0.0352 (14)	0.0650 (19)	0.0015 (11)	0.0236 (14)	0.0066 (12)
C6	0.0362 (13)	0.0276 (12)	0.0517 (15)	0.0044 (10)	0.0190 (12)	0.0039 (10)
C7	0.0386 (13)	0.0340 (13)	0.0480 (15)	0.0036 (11)	0.0216 (12)	0.0056 (11)
C8	0.0364 (12)	0.0302 (12)	0.0347 (12)	0.0023 (10)	0.0172 (10)	0.0055 (9)
C9	0.0321 (12)	0.0359 (13)	0.0291 (12)	0.0045 (10)	0.0121 (10)	0.0036 (9)
C10	0.0336 (12)	0.0361 (13)	0.0319 (12)	0.0035 (10)	0.0151 (10)	−0.0036 (10)
C11	0.0459 (14)	0.0335 (13)	0.0340 (13)	−0.0012 (11)	0.0163 (11)	−0.0043 (10)
C12	0.0546 (16)	0.0338 (14)	0.0499 (16)	−0.0018 (12)	0.0235 (14)	−0.0036 (11)
C13	0.0499 (16)	0.0396 (15)	0.0576 (17)	−0.0069 (12)	0.0245 (14)	−0.0101 (13)
C14	0.0425 (15)	0.0511 (17)	0.0470 (16)	−0.0064 (13)	0.0132 (12)	−0.0118 (13)
C15	0.0383 (13)	0.0471 (15)	0.0357 (13)	0.0031 (11)	0.0131 (11)	−0.0022 (11)
C16	0.0406 (14)	0.0395 (14)	0.0545 (17)	0.0083 (12)	0.0190 (13)	0.0072 (12)
C17	0.0347 (12)	0.0273 (11)	0.0312 (12)	−0.0021 (9)	0.0129 (10)	−0.0013 (9)
C18	0.0363 (13)	0.0554 (17)	0.0446 (15)	0.0087 (12)	0.0172 (12)	0.0066 (12)

### Geometric parameters (Å, º)

**Table:**

O1—C7	1.227 (3)	C9—C10	1.522 (3)
O2—C17	1.245 (3)	C9—H9	1.0000
N1—C17	1.336 (3)	C10—C11	1.393 (3)
N1—C9	1.473 (3)	C10—C15	1.394 (3)
N1—H1N	0.94 (3)	C11—C12	1.396 (4)
C1—C6	1.390 (3)	C11—H11	0.9500
C1—C2	1.392 (4)	C12—C13	1.385 (4)
C1—H1	0.9500	C12—H12	0.9500
C2—C3	1.386 (4)	C13—C14	1.380 (4)
C2—H2	0.9500	C13—H13	0.9500
C3—C4	1.381 (4)	C14—C15	1.396 (4)
C3—H3	0.9500	C14—H14	0.9500
C4—C5	1.380 (4)	C15—H15	0.9500
C4—H4	0.9500	C16—H16A	0.9800
C5—C6	1.407 (3)	C16—H16B	0.9800
C5—H5	0.9500	C16—H16C	0.9800
C6—C7	1.494 (4)	C17—C18	1.505 (3)
C7—C8	1.531 (3)	C18—H18A	0.9800
C8—C16	1.538 (3)	C18—H18B	0.9800
C8—C9	1.538 (3)	C18—H18C	0.9800
C8—H8	1.0000		
			
C17—N1—C9	123.2 (2)	C8—C9—H9	108.2
C17—N1—H1N	117.8 (16)	C11—C10—C15	118.4 (2)
C9—N1—H1N	119.0 (16)	C11—C10—C9	120.5 (2)
C6—C1—C2	120.8 (3)	C15—C10—C9	121.0 (2)
C6—C1—H1	119.6	C10—C11—C12	120.6 (2)
C2—C1—H1	119.6	C10—C11—H11	119.7
C3—C2—C1	119.6 (3)	C12—C11—H11	119.7
C3—C2—H2	120.2	C13—C12—C11	120.2 (3)
C1—C2—H2	120.2	C13—C12—H12	119.9
C4—C3—C2	120.6 (3)	C11—C12—H12	119.9
C4—C3—H3	119.7	C14—C13—C12	119.7 (3)
C2—C3—H3	119.7	C14—C13—H13	120.2
C5—C4—C3	119.6 (3)	C12—C13—H13	120.2
C5—C4—H4	120.2	C13—C14—C15	120.2 (3)
C3—C4—H4	120.2	C13—C14—H14	119.9
C4—C5—C6	121.1 (3)	C15—C14—H14	119.9
C4—C5—H5	119.4	C10—C15—C14	120.8 (3)
C6—C5—H5	119.4	C10—C15—H15	119.6
C1—C6—C5	118.2 (2)	C14—C15—H15	119.6
C1—C6—C7	123.0 (2)	C8—C16—H16A	109.5
C5—C6—C7	118.9 (2)	C8—C16—H16B	109.5
O1—C7—C6	120.4 (2)	H16A—C16—H16B	109.5
O1—C7—C8	119.9 (2)	C8—C16—H16C	109.5
C6—C7—C8	119.6 (2)	H16A—C16—H16C	109.5
C7—C8—C16	107.2 (2)	H16B—C16—H16C	109.5
C7—C8—C9	110.49 (19)	O2—C17—N1	122.5 (2)
C16—C8—C9	111.9 (2)	O2—C17—C18	121.0 (2)
C7—C8—H8	109.1	N1—C17—C18	116.5 (2)
C16—C8—H8	109.1	C17—C18—H18A	109.5
C9—C8—H8	109.1	C17—C18—H18B	109.5
N1—C9—C10	111.73 (19)	H18A—C18—H18B	109.5
N1—C9—C8	108.67 (19)	C17—C18—H18C	109.5
C10—C9—C8	111.73 (18)	H18A—C18—H18C	109.5
N1—C9—H9	108.2	H18B—C18—H18C	109.5
C10—C9—H9	108.2		
			
C6—C1—C2—C3	0.8 (4)	C7—C8—C9—N1	−58.1 (2)
C1—C2—C3—C4	−0.1 (4)	C16—C8—C9—N1	−177.54 (19)
C2—C3—C4—C5	−0.8 (4)	C7—C8—C9—C10	178.12 (19)
C3—C4—C5—C6	1.0 (4)	C16—C8—C9—C10	58.7 (3)
C2—C1—C6—C5	−0.5 (4)	N1—C9—C10—C11	−47.8 (3)
C2—C1—C6—C7	178.6 (2)	C8—C9—C10—C11	74.1 (3)
C4—C5—C6—C1	−0.4 (4)	N1—C9—C10—C15	133.1 (2)
C4—C5—C6—C7	−179.5 (2)	C8—C9—C10—C15	−104.9 (2)
C1—C6—C7—O1	−174.8 (2)	C15—C10—C11—C12	−0.1 (3)
C5—C6—C7—O1	4.3 (4)	C9—C10—C11—C12	−179.2 (2)
C1—C6—C7—C8	2.8 (3)	C10—C11—C12—C13	−1.1 (4)
C5—C6—C7—C8	−178.0 (2)	C11—C12—C13—C14	1.3 (4)
O1—C7—C8—C16	86.0 (3)	C12—C13—C14—C15	−0.3 (4)
C6—C7—C8—C16	−91.6 (3)	C11—C10—C15—C14	1.0 (4)
O1—C7—C8—C9	−36.1 (3)	C9—C10—C15—C14	−179.9 (2)
C6—C7—C8—C9	146.2 (2)	C13—C14—C15—C10	−0.9 (4)
C17—N1—C9—C10	−102.4 (2)	C9—N1—C17—O2	3.4 (3)
C17—N1—C9—C8	133.9 (2)	C9—N1—C17—C18	−176.2 (2)

### Hydrogen-bond geometry (Å, º)

Cg1 and Cg2 are the centroids of the C10–C15 and C1–C6 benzene rings, respectively.

**Table:**

*D*—H···*A*	*D*—H	H···*A*	*D*···*A*	*D*—H···*A*
N1—H1*N*···O2^i^	0.94 (3)	1.98 (3)	2.874 (3)	158 (2)
C1—H1···*Cg*1^i^	0.95	2.85 (1)	3.649 (3)	142 (1)
C16—H16*A*···*Cg*2^ii^	0.98	2.98 (1)	3.472 (3)	112 (1)

Symmetry codes: (i) *x*, −*y*+3/2, *z*−1/2; (ii) −*x*+1, *y*+3/2, −*z*+3/2.
